# Inkjet Printed Y-Substituted Barium Zirconate Layers as Electrolyte Membrane for Thin Film Electrochemical Devices

**DOI:** 10.3390/membranes9100131

**Published:** 2019-10-11

**Authors:** Theodor Schneller, David Griesche

**Affiliations:** Institut für Werkstoffe der Elektrotechnik 2 & JARA-FIT, RWTH Aachen University, 52074 Aachen, Germany

**Keywords:** inkjet printing, chemical solution deposition, BZY, proton conductors, thin films, additive manufacturing, IT-SOFCs

## Abstract

In this work, the inkjet printing of proton conducting Y-substituted barium zirconate (BZY) thin films was studied. Two different kinds of precursor inks, namely a rather molecular BZY precursor solution and a BZY nanoparticle dispersion, have been synthesized and initially investigated with regard to their decomposition and phase formation behavior by thermal analysis, X-ray diffraction, and scanning electron microscopy. Their wetting behavior and rheological properties have been determined in order to evaluate their fundamental suitability for the inkjet process. Crystalline films have been already obtained at 700 °C, which is significantly lower compared to conventional solid-state synthesis. Increasing the temperature up to 1000 °C results in higher crystal quality. Permittivity measurements gave values of around 36 that are in good agreement with the literature while also proving the integrity of the materials. A modification of the as-synthesized BZY stock solution and nanoparticle dispersion by dilution with propionic acid improved the jetability of both inks and yielded homogeneous BZY coatings from both inks. In order to study the electrochemical properties of BZY films derived from the two printed inks, BZY coatings on sapphire substrates were prepared and characterized by electrochemical impedance spectroscopy.

## 1. Introduction

Conventional solid oxide fuel cells (SOFCs) based on oxide ion conducting electrolytes such as yttrium stabilized zirconia (YSZ) are typically operated in the range 800–1000 °C and are therefore denoted as high-temperature (HT) solid oxide fuel cells [1,2]. These high temperatures are causing a number of issues such as the need for expensive materials for cell interconnectors, thermal stresses, possible interface reactions, comparatively long heat-up times, and high energy input for this heat-up phase. Thus, there is a quest to lower these temperatures down to the so-called intermediate temperature (IT) range of approximately 400–700 °C [3,4,5,6,7]. Typical approaches to achieve this aim are based on a reduction of the electrolyte layer thickness and/or an optimization of the electrolyte material properties itself. This applies not only therefore, but also during the implementation of SOFCs in miniaturized devices, i.e., µ-SOFCs [8], thin film (It has to be mentioned that the definition of what is a thin film is sometimes contradictory and depends a bit on the community of the corresponding research group, i.e., for classical ceramic technology a level of, thickness down to ~1 µm is often called a “thin film” while for groups working in the field of integrated devices, thin films are typically far below 1 µm down to some tens of nanometers. According to a common definition, 1 µm are denoted as thin films and thicknesses above 1 µm are denoted as thick films. In the present work, typical thin films are used.) electrolytes are gaining increasing significance. However, it should be mentioned that in the case of low operation temperatures, the electrodes performance, and in particular the cathode activity due to the overpotential of the oxygen processes (e.g., reduction and transport) has to be considered as well [9]. Concerning the optimization of the electrolyte material, the main research activities were directed for years towards oxygen ion conductors beyond the classical YSZ, such as gadolinium doped ceria (CGO) [6,7,10,11,12]. However, high-temperature proton conducting metal oxide materials (high-temperature protonic conductors - HTPCs) became increasingly useful for the development of IT-SOFCs due to a number of advantages, such as lower activation energies for the proton (~0.3–0.6 eV) [4,13,14,15,16,17,18,19] compared to oxygen ion (~0.6–1.2 eV) [20,21] transport, leading to higher conductivities in the IT range. In addition, there is no dilution of the fuel since the water vapor evolution occurs on the oxygen side of the cell [9,20,22]. Perovskite electrolytes based on BaCeO_3_ (BCO), BaZrO_3_ (BZO), and solid solutions thereof that have been B-site doped with trivalent cations such as Y^3+^, i.e., BaCe_(1−x−y)_Zr_x_Y_y_O_3−δ_ (typically 0 < x < 0.9; 0.1 < y < 0.2), represent the probably most frequently investigated HTPCs. While acceptor doped BCO shows excellent proton conductivities up to ~10^−2^ S/cm at 600 °C, its chemical stability under acid gases is rather poor and leading to decomposition, e.g., into BaCO_3_ in the presence of CO_2_ [4]. On the other hand, acceptor doped BZO is chemically stable and has a high bulk conductivity [22,23], however this causes difficulties due its refractory nature such as meager sinterability, leading to smaller grains and about one order of magnitude lower total proton conductivity in sintered ceramic pellets due to the poor grain boundary conductance [4]. The properties of the studied acceptor doped solid solutions of BCO and BZO are in-between and often represent a good compromise. In summary Y-doped BZO represents one of the most promising HTPC materials studied for IT-SOFC and large research effort is directed to understand the physical and structural reasons for the low overall conductance of the ceramics and finding approaches to optimize them [4,5,13,24,25,26,27,28,29,30,31,32].

Furthermore it is expected that the combination of HTPCs with genuine thin film techniques such as pulsed laser deposition (PLD) [33,34,35,36,37], atomic layer deposition (ALD) [38,39], or chemical solution deposition (CSD) [40,41] can further contribute to the optimization of the conductivities of HTPCs. Among the available thin film fabrication techniques, CSD offers a number of advantages, such as easy control over stoichiometry and relatively low investment cost [42]. Often the intimate mixing of the individual precursor components on a molecular level leads to significantly lower crystallization temperatures compared to typical bulk ceramic processing, which is also beneficial for reducing destructive influences at interface regions during material processing. The most straightforward wet chemical thin film preparation method is spin coating of a suitable precursor solution onto the substrate with subsequent thermal processing leading to coatings over the full area. For device manufacturing, subtractive methods based on photolithography and etching normally have to be applied to pattern the deposited functional film. This requires a number of additional processing steps and a possible deteriorating influence of the etching step on underlying materials cannot be easily ruled out. Hence, additive or direct writing methods, which enable simultaneous deposition and patterning of (metal oxide) functional thin films, are attracting considerable interest [43]. These methods include inkjet printing, which was originally introduced to print text and graphics from a computer (PC), and evolved into a powerful tool for materials and device manufacturing. Starting with the metallization of solar cells [44,45,46], more and more application fields were made accessible. They range from printable solar cells [47] and (organic) electronics including metallization [48,49,50,51,52,53,54,55] to various functional metal oxide films such as coated superconductors (buffer layer [56,57] and the superconducting layer itself [58,59,60,61]), ferro- and piezo-electric layers [62,63,64], dielectric layers [65,66,67,68], transparent conducting oxides [69,70], etc. Their general advantages include less waste production, non-contact technique and easy digital computer-aided pattern generation, modification, and storage. The possibility of a continuous deposition in a reel-to-reel approach as required in the case of coated conductors is an additional benefit [71]. For many materials, metallo-organic precursor solutions and metal oxide (nano)particle dispersions represent the ink of choice. Depending on the humidity sensitivity of the involved precursor solutions, the CSD routes can be roughly categorized into the stable metallo-organic decomposition (MOD), unstable sol-gel, and a hybrid which resides between the two types [42]. Drop on demand (DOD) inkjet printing systems based on piezoelectric actuation are mainly employed, although thermal (bubble jet) and electromagnetic systems can be used as well depending on the aimed device and the chemistry of the used inks. However, if sol-gel types of precursors are utilized, care has to be taken to avoid moisture, and thermal systems usually do not work well in this case due to the accelerated sol-gel transition in the nozzles causing clogging.

While a considerable number of works have been published in the different research fields mentioned above, only a few papers describe the inkjet deposition of electrolyte and electrode materials for SOFCs [72,73,74,75,76,77,78]. Moreover, until now these studies have only dealt with O^2−^-ion conducting film electrolytes based on YSZ or CGO in the thickness range > 1 µm, i.e., thick films, and most of them use suspended ceramic particles as inks for the deposition. To the best of the author’s knowledge, no works have been published yet concerning inkjet printing of HTPC thin films.

Hence in the present work the fabrication of full area coated thin film proton conducting electrolytes based on yttrium substituted barium zirconate (Ba(Zr_0.9_Y_0.1_)O_3−δ_-BZY10) by inkjet printing is investigated. The printing and phase formation behavior of two different kinds of ink, namely rather molecular precursor solutions and reverse micelle stabilized nanoparticle dispersions, is studied. This comprises specific aspects of this deposition technique as well as the morphological and electrochemical characterization of the prepared dense BZY10 films.

## 2. Materials and Methods

### 2.1. Ink-Syntheses

Two different precursor inks have been synthesized. *Ink-*1 corresponds to a more molecular or small oligomeric species containing solution obtained by dilution of a conventional precursor solution (*con*-BZY10) with propionic acid, while *ink-*2 refers to an accordingly diluted nanoparticle based dispersion (*µE*-BZY10). The composition of both types of precursor inks was Ba(Zr_0.9_Y_0.1_)O_3−δ_ (BYZ10) and the obtained stock solutions were stored and diluted freshly prior to the printing experiments.

The synthesis of 50 mL *con*-BZY10 (*ink-*1) was divided in two steps by preparing two partial solutions separately. At first 2.96 g high purity barium carbonate (Alfa Aesar, Puratronic^®^, 99.997%, Kandel, Germany) was weighed in and the corresponding amount (0.499 g) of yttrium acetate hydrate (Alfa Aesar, 99.9%) was added. After adding 15 mL propionic acid (Alfa Aesar, 98%) and 3 mL of propionic acid anhydride (Sigma-Aldrich, 97%, Darmstadt, Germany) to remove the water of crystallization, the suspension was refluxed at 140 °C for approximately 2 h under argon until a clear solution was obtained. Subsequently this solution was concentrated by distillation under reduced pressure. The resulting viscous mixture was re-dissolved in a few milliliters of propionic acid to form partial solution 1.

Partial solution 2 was prepared in a glovebox by addition of two equivalents (2.803g [2.89 mL]) of 2,4-pentanedione (Hacac; Fluka, 99.5%) to the solution of 6.182 g zirconium tetrabutoxide (TBZ; Alfa Aesar, 80% w/w in 1-butanol) in ~ 23 mL n-butanol (*n*-BuOH; Sigma-Aldrich, 99.5%). Next, the glovebox partial solution 1 was quantitatively transferred to partial solution 2. Finally, the concentration of the stock solution was adjusted to 0.3 mol/L by adding propionic acid. After filtering through a 0.2 µm PTFE filter, this solution could be basically used for coating experiments. However in order to improve the jetting behavior and to reduce the risk of blocking the nozzles, for the printing experiments this stock solution was diluted with propionic acid in a ratio of 1:1. Less good printing results were obtained by dilution with *n*-BuOH.

Reverse micelles of the type “*water in oil*” were used as nano-reactors to synthesize BZY10 nanoparticles dispersions (*ink-*2). The corresponding microemulsion (*µE*) was fabricated from 3.74 wt% cetyltrimethylammonium bromide (CTAB) as cationic surfactant, 12.21 wt% 1-pentanol (co-surfactant), and 72.82 wt% cyclohexane (oil-phase) as described earlier [79]. It contained 11.23 wt% ultrapure degassed and argon saturated water.

As a moisture sensitive precursor for the nano scaled sol-gel process, a mixed metallo-organic Ba-Zr-Y alkoxide compound was prepared by dissolving 2.5453 g barium-metal in methanol, and adding 7.1411 g zirconium tetra *n*-propoxide (76.5 wt% in n-propanol) and 1.9733 g yttrium tri *iso*-propoxide (25 wt% in toluene) to the reaction mixture. In order to accomplish the synthesis procedure a stoichiometric amount (8.7637 g) of the microemulsion was added dropwise at room temperature. This leads to the formation of amorphous BZY10 nanoparticles which are stabilized by the surfactant molecules absorbed on the surface. A stable dispersion with a solid content of 5 wt% corresponding to ~0.15 mol L^−1^ was obtained. For the printing experiments, the BZY nanoparticle dispersion was diluted 1:1 with propionic acid in order to improve the rheology for the printing process. Experiments with methanol as solvent for dilution were less controllable due to the high vapor pressure of this solvent at room temperature.

### 2.2. Printer and Parameters

For ink-jet printing of BZY precursor layers, a *Pixdro LP50 DOD* (Drop-On-Demand) print system equipped with a *Spectra S-Class* print head was used. This print head has 128 single line arranged nozzles that have a distance of 508 µm between each other. The individual nozzles have a diameter of 35 µm. According to the manufacturer, this print head can be used with basic and acid solvents. The head and substrate holder can be basically aligned in x-, y- and z- directions in the Cartesian coordinate system and moreover two angles can be defined. The specified drop-size range is 25–30 picoliters. For the drop view analysis *ink-*1, 100 V, and 12 µs pulse length, and a pressure in the ink reservoir of −18.5 mbar were applied. *Ink-*2 was operated with 100 V, 13 µs pulse length, and −16 mbar.

### 2.3. Film Processing

All films have been subjected to a crystallization anneal in a diffusion furnace under oxygen atmosphere directly after each individual deposition step, irrespective of the type of coating method (i.e., inkjet or spin-on process). If not otherwise indicated, a crystallization temperature of 1000 °C was applied. In order to adjust the final thickness, multiple deposition and annealing steps were applied. In case of spin coating a spinning speed of 3000 RPM was used for all samples derived from molecular precursor solutions (*con*-BZY10), while for those obtained from nanoparticle dispersions (*µE*-BZY10), 4000 RPM were employed.

### 2.4. Analysis

The viscosities *η* of the different inks have been determined with a *Brookfield DV-II+* viscosimeter at 25 °C and t.

The film morphology was investigated by scanning electron microscopy (SEM) using a *Zeiss DSM 982 Gemini* instrument. Specimen have been prepared for cross-section analysis by braking and gluing them on an inclined sample holder by conductive silver paste.

The surface topography of the substrates was characterized using a commercial atomic force microscope (AFM; Nanosurf Flex Axiom, Switzerland) at ambient conditions in tapping mode using n^+^-doped silicon cantilevers (Nanosensors). RMS analysis was performed after plane correction on an area of 25 µm^2^ by applying Gwyddion.

The X-ray diffraction (XRD) pattern for the study of the phase evolution was recorded with a *Panalytical X’Pert Pro* system with Cu K_α_ radiation, *λ* = 1.5405 Å.

DTA-TG analyses (differential thermo analysis coupled with thermo gravimetry - *Bähr STA* 502) were carried out to study the decomposition behavior of the prepared precursor inks. Prior to the measurement, the major part of the solvents was first removed by distillation from the precursor solutions and BZY nanoparticle dispersions at moderate temperatures (max. 70 °C) under reduced pressure in order to prepare a suitable specimen for analysis. Afterwards, 62.69 mg of the *con*-BZY10 and 66.32 mg of *µE*-BZY10 precursor gels were weighed into alumina crucibles and subjected to heating under air with a constant rate of 5 K/min up to 1000 °C. A comparable amount of Al_2_O_3_ was used as the reference material.

The contact angle and wettability of the two precursor inks on different substrate surfaces were investigated with a Krüss drop shape analyzer *DSA100B*. For measuring the surface tension *γ* of the two inks, a DataPhysics – OCA20 all-purpose measuring device for contact angle measurements and drop shape analysis was used, as was t.

The densities *ρ* of the two precursor inks were determined by weighing calibrated flasks tempered at 25 °C with a volume of 2 mL.

The nanoparticle size distribution was determined by the method of dynamic light scattering (DLS) using a Malvern *Zetasizer Nano S* instrument.

For the high temperature electrochemical impedance spectroscopy (HT-EIS), a commercial system (Novotherm HT 1200, equipped with an Alpha-A analyzer with Pot/Gal, Novocontrol GmbH, Germany) was utilized. The entire construction including a temperature sensor could be moved into the vertical oven. Measurements were performed in a two-electrode in-plane setup. The required samples were prepared on sapphire. Commercially available C-cut (0001) substrates were used for the film deposition by inkjet printing. After crystallization, two rectangular 200 nm thick Pt-electrodes (length L = 5 mm, width B = 3 mm) with a nominal distance D of 1 mm (sample design see Section 3.4) were deposited by sputtering and lithographic lift-off processing. Since the required undercut resist pattern was prepared by using a foil mask, there was an uncertainty in the final electrode distance. Hence, for the calculation of the conductivity, the real dimensions measured at the final Pt top electrodes have been used.

The electrodes were contacted with platinum wires and the HT-EIS measurements were performed in humidified nitrogen atmosphere (p(H_2_O) ~31 mbar). For the latter, nitrogen was bubbled through water filled washing flasks at room temperature. The samples were analyzed with an alternating voltage of *U_~_* = 10 mV in the frequency range of 1 MHz–1 Hz. Measurements were carried out in a temperature range of 600–150 °C and a cooling ramp with 50 °C steps. A dwell time of one hour after each temperature step was applied to allow for thermal and atmospheric equilibration of the sample.

## 3. Results and Discussion

In order to generate functional HTPC electrolytes for application in IT-SOFCs by using inkjet printing, a number of parameters and processing steps have to be controlled properly. Thus, all the relevant steps beginning with the chemistry of the precursor inks and their fundamental phase formation behavior followed by the study of the parameters required for the print process itself up to the morphological and electrochemical characterization will be described in the following sections.

### 3.1. Precursor Systems

Two chemically different kinds of BZY10 precursor systems denoted as *con*-BZY10 and *µE*-BZY10 have been prepared for the printing experiments in this work. While *con*-BZY10 represents a conventional BZY10 precursor solution based on rather molecular educts of three metallo-organic compounds that are dissolved in a common solvent, *µE*-BZY10 consists of a reverse micelle stabilized BZY10 nanoparticle dispersion.

**Figure 1:**
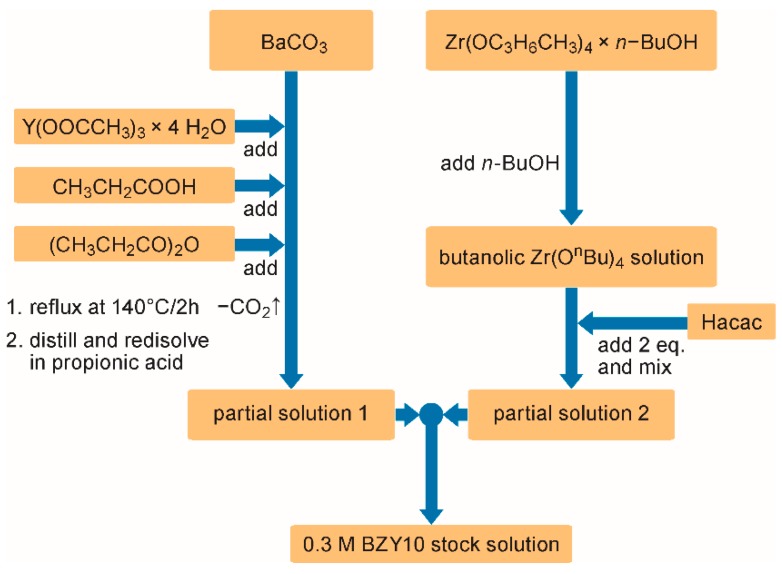
Flow chart of the propionate based conventional BZY10 solution synthesis.

The synthesis of the 0.3 M *con*-BZY10 stock solution is based on a modified and optimized route described in reference [41]. Briefly, barium carbonate and yttrium acetate were refluxed in a mixture of propionic acid and propionic acid anhydride until a clear solution is formed (Figure 1). After reducing the volume of this solution and refilling it with propionic acid, the resulting “partial solution 1” was added to “partial solution 2” which consisted of TBZ stabilized with two equivalents of Hacac in *n*-butanol (Figure 1).

**Figure 2:**
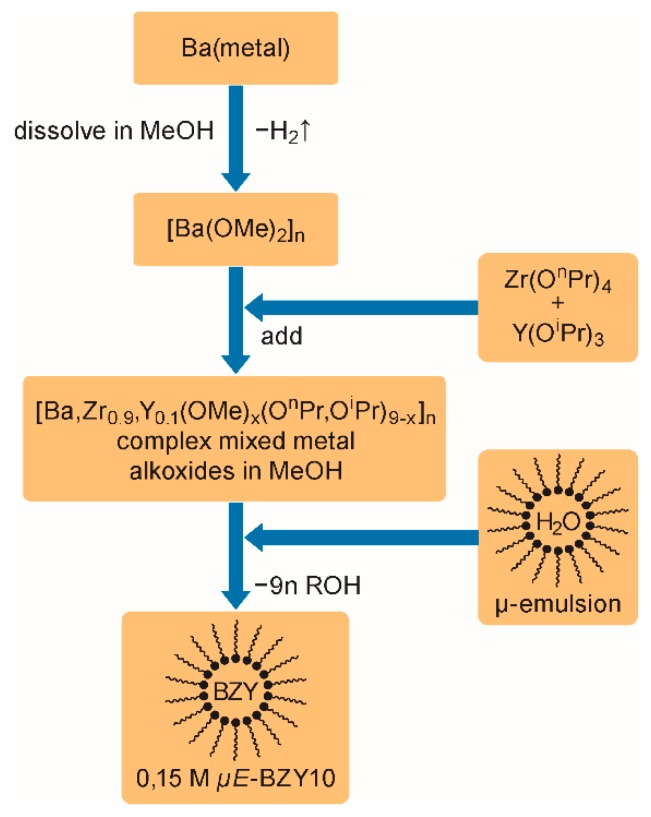
Synthesis procedure for BZY10 nanoparticle dispersions. (It should be noted that the indicated stoichiometry of the complex mixed metal alkoxide is idealized, and is the only basis for the calculation of the required amount of water in the form of the micro emulsion).

Figure 2 schematically shows the preparation of the *µE*-BZY10 stock dispersion. The procedure is performed in analogy to earlier work for reverse micelle based barium titanate nanoparticle dispersions [79]. At first, barium metal is dissolved in anhydrous methanol followed by the addition of a Zr/Y *n*/*iso*-propoxide mixture in the required 9:1 ratio. The resulting dispersion of complex mixed metal alkoxides is subjected to a quantitative sol-gel reaction within the nanometer sized water droplets of the reversed micelles formed from CTAB (see reference [79]). Finally, a sTable 5 wt% BZY10 nanoparticle containing dispersion corresponding to a concentration of ~0.15 mol L^−1^ was obtained.

**Figure 3:**
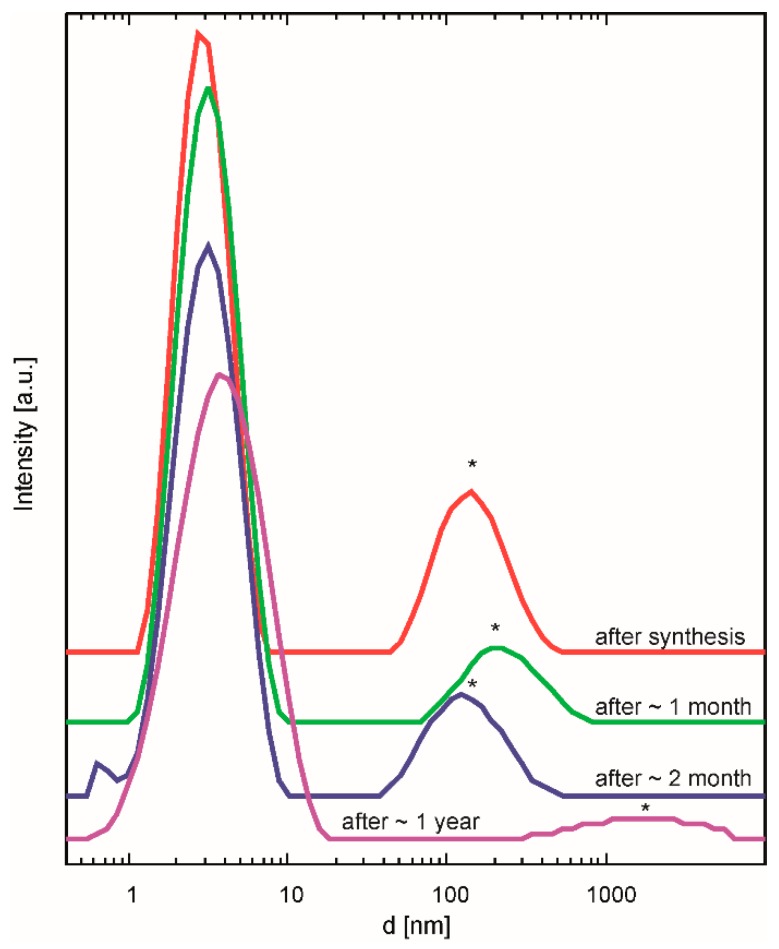
DLS measurements of *µE*-BZY10 after different times of ageing as indicated. The second peaks marked with asterisks were attributed to apparent agglomerates caused by the concentration.

In order to characterize the essential properties of these two stock solutions, dynamic light scattering (DLS) experiments, thermal analyses, contact angle measurements, and the study of phase formation were performed. From the DLS measurements, the size of the BZY10 nanoparticles in *µE*-BZY10 was found to be around 3–4 nm (Figure 3). The ageing of the dispersions was monitored by repeated DLS measurements after different times. Up to one year afterwards, no significant change in the average particle size could be detected, indicating an excellent stability of the dispersion (Figure 3). Since the dispersion was filtered through a 200 nm pore size PTFE filter before the measurement, the larger particles found in the measurements were attributed to apparent agglomerates. They practically vanish if the initial *µE*-BZY10 dispersion is diluted with a suitable solvent (e.g., with methanol in a 1:1 ratio) as shown exemplarily in the measurement “after 1 year” in Figure 3. Similar results were obtained by dilution with propionic acid (not shown) which leads to a better printing performance, as will be described below (Section 3.2).

#### 3.1.1. Decomposition Behavior

**Figure 4:**
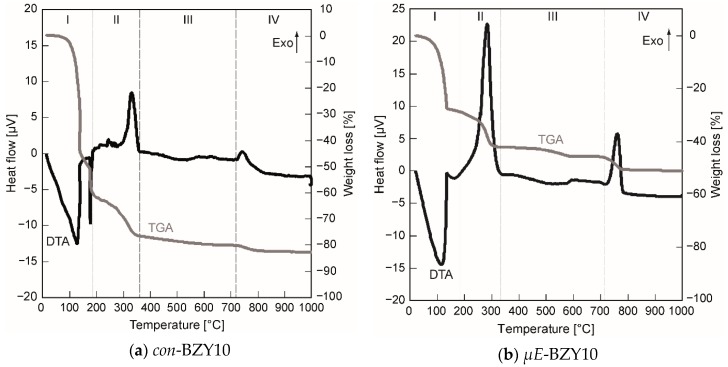
Differential thermo analysis coupled with thermo gravimetry (DTA/TG) analysis of the molecular (**a**) and the BZY10 nanoparticle (**b**) based precursor systems. The four different indicated regions (I–IV), corresponding to evaporation (I), exothermic decomposition (II), no pronounced reaction (III), and crystallization (IV) are discussed in detail in the text.

In order to figure out suitable temperature ranges for the thermal annealing of the BZY films, the decomposition of the organic precursor ingredients and the onset of crystallization of both precursor inks were studied by DTA/TG. Figure 4 shows the results of these measurements.

Four regions can be identified in both diagrams (Figure 4). In region I, endothermic evaporation of solvents entrapped in the precursor gels takes place (Figure 4a—n-butanol {b.p. 118 °C} and propionic acid {b.p. 141 °C}; Figure 4b—cyclohexane {b.p. 81 °C} and 1-pentanol {b.p. 138 °C}). Region II corresponds to exothermic decomposition of residual organic material, in region III which corresponds predominantly to the X-ray amorphous metal oxides, no significant reactions and moderate weight losses occurs. Finally, region IV is initiated by the onset of crystallization accompanied with a minor mass loss until the constant weight of the crystalline BZY10 powder is achieved. This final mass loss, 5.3% in case of *µE*-BZY and 2.3% in case of *con*-BZY (Figure 4), is attributed to the release of carbon dioxide stemming from the decomposition of residual barium carbonate species during the crystallization process. Anyway, this is only a quite low BaCO_3_ content compared to a hypothesized situation with an equimolar BaCO_3_ formation in the amorphous material. Here one would expect a considerably higher weight loss of 13.7% due to the CO_2_ release.

Although the basic course of the decomposition is similar to both precursor solutions, some interesting features can be figured out. In case of the *µE*-BZY dispersion, the evaporation and the decomposition of organic ligands was already finished at slightly lower temperatures compared to the *con*-BZY solution and accompanied by a reduced weight loss of Δm ~ −14% for *µE*-BZY compared to ~ −19% for *con*-BZY in the decomposition phase II in Figure 4. This can be explained in terms of a lower content of non-evaporable organics in the BZY-nanoparticle dispersion caused by the ideally stoichiometric removal of alkoxy ligands during the sol-gel reaction within the reverse micelles. The last significant exothermic decomposition peak in this phase at ~280 °C (Figure 4b) may be explained by the combustion of CTAB. In contrast, this decomposition step is found ~ 50 °C above (332 °C) for the *con*-BZY solution (Figure 4a). Thus, it might be expected that a lower crystallization temperature of the *µE*-BZY derived material can be obtained, but in fact the crystallization temperature is actually slightly higher (765 °C vs. 743 °C in Figure 4). The reason for this could be the slightly higher content of barium carbonate in the *µE*-BZY derived powder formed by the reaction of barium oxide with carbon dioxide taken up from the ambient during the initial phase of the heating experiment. Barium carbonate is known to be very stable up to high temperatures and thus delays the crystallization process. Nevertheless if an annealing temperatures above 765 °C is applied, which is much lower than in corresponding solid state syntheses (typically around ~1500 °C, see reference [4] for an example), then crystalline BZY10 powders should be obtained. For thin films this could even be a bit lower due to decomposition and nucleation promoting effects of the substrate. Actually, in the case of both coating solutions having already reached 700 °C, clearly crystalline BZY10 thin films can be obtained from spin coated solutions after annealing in a diffusion furnace in oxygen atmosphere (see Section 3.1.3).

#### 3.1.2. Wetting Behavior

**Figure 5:**
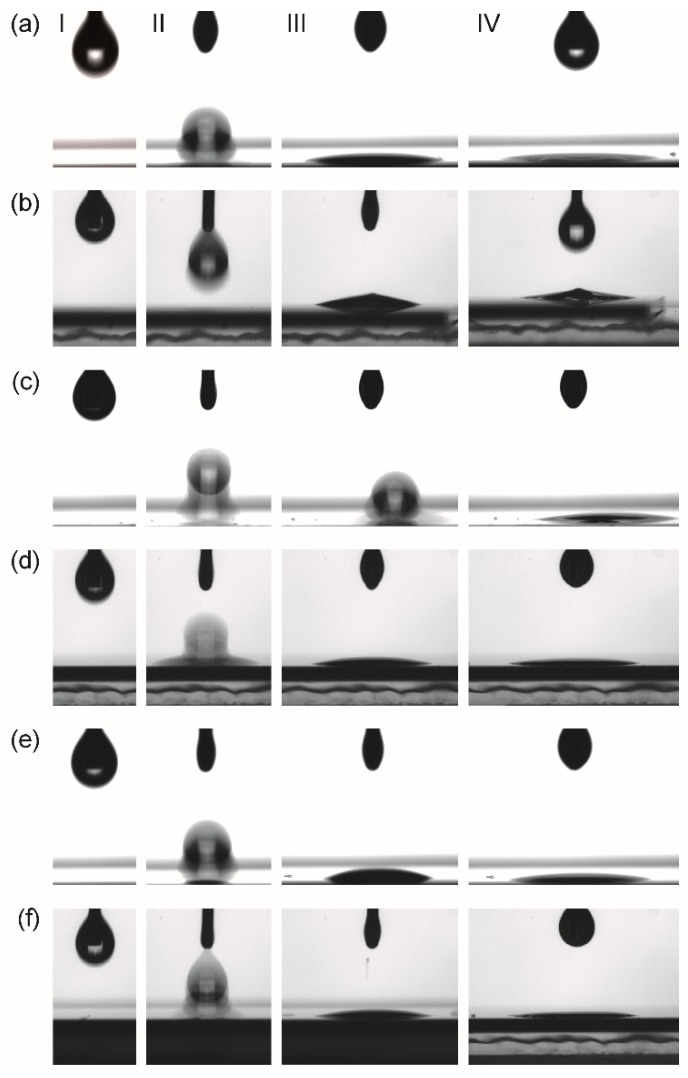
Images of characteristic stages (I–IV) of the contact angle measurements extracted from the movie clips recorded from the *Drop Shape Analyzer* instrument. I: start of experiment; II: impact of the droplet on the surface; III: situation after 1-2 s after impact. IV: just before impact of next droplet. (**a**) *con*-BZY and (**b**) *µE*-BZY on platinized silicon substrates; (**c**) *con*-BZY and (**d**) *µE*-BZY on fully BZY10 coated platinized silicon substrates; (**e**) *con*-BZY and (**f**) *µE*-BZY on oxidized silicon substrates. It should be noted that in the case of *con*-BZY droplet deposition onto BZY in (**c**), there is a slight movement of the droplet to the off-axis position. A possible reason for this could be an accidental impurity on the surface at the position of the droplet impact.

Suitable wetting of substrate surfaces is of general importance for film deposition from liquid phases. For laminar coatings of complete wafer areas by spin or dip coating, a complete easy spreading (contact angle θ ~ 0°) of the as-deposited precursor solution is ideal. However, for ink-jet printing, a good wettability is needed rather than complete spreading, meaning enabling a certain resolution of the printed pattern is desirable. To elucidate the wetting properties of the precursor systems used in this work contact angle analyses of deposited droplets on different surfaces have been performed. Three different surfaces, namely platinized silicon, oxidized silicon, and BZY10 coated platinized silicon were chosen. The latter interface is important due to the multiple coating steps typically necessary in CSD processing for achieving a certain final film thickness. The results of these measurements are summarized in Figure 5.

Even though the contact angle on all substrates was too low to be measured with the instrument (Figure 5), it can be concluded that it is close to zero. Thus, good coating properties can be expected for all combinations of precursors and surfaces investigated in this work.

#### 3.1.3. Phase Formation

**Figure 6:**
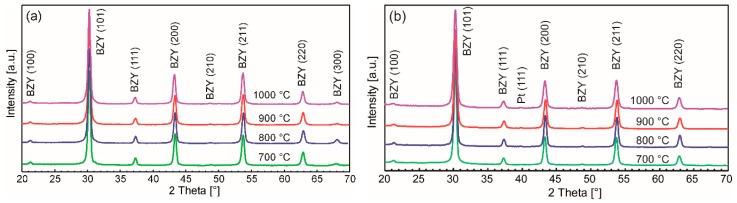
XRDs of BZY10 films derived from the two precursor systems. (**a**) *con*-BZY and (**b**) *µE*-BZY annealed at different temperatures recorded in grazing incidence. Both films show already the expected reflections for crystalline BZY at 700 °C with cubic indexation. Temperatures and reflex indexes are indicated in the diagrams.

The phase formation behavior was elucidated by crystallization experiments of spin coated precursor films derived from the two different precursor systems in the temperature range from 700 –1000 °C. The corresponding X-ray diffraction (XRD) patterns are shown in Figure 6.

Barium zirconate (BZO) as the parent material exhibits a cubic perovskite structure with a lattice constant of 4.193 Å (JCPDS file #06-0399) in the bulk. After conducting a partial substitution of the Zr^4+^ with the larger Y^3+^ cation, a linear increase of the lattice constant is expected. Although in some works a slight lattice distortion for BZY10 bulk materials has been observed [13,80], most studies deduce a cubic structure from the XRDs with a lattice constant ranging from 4.20 to 4.209 Å [15,81,82,83,84,85,86,87]. A good summary of the different published constants for BZY10 may be found in reference [88]. In accordance with these studies and due to the absence of peak splitting of the diffraction peaks, the BZY10 thin films in this work have been indexed as being cubic as well. However, the calculation of the lattice constants from the measurements shown above lead to somewhat lower values of ā = 4.175 Å which might be explained by compressive stress in the BZY10 thin films caused by the different thermal expansion coefficients of substrate and the clamped electrolyte film. A comparative deposition of an undoped BZO film by the analogue precursor chemistry and the same process also yielded a lower lattice parameter of 4.170 Å (XRD not shown) compared to bulk BZO.

**Figure 7:**
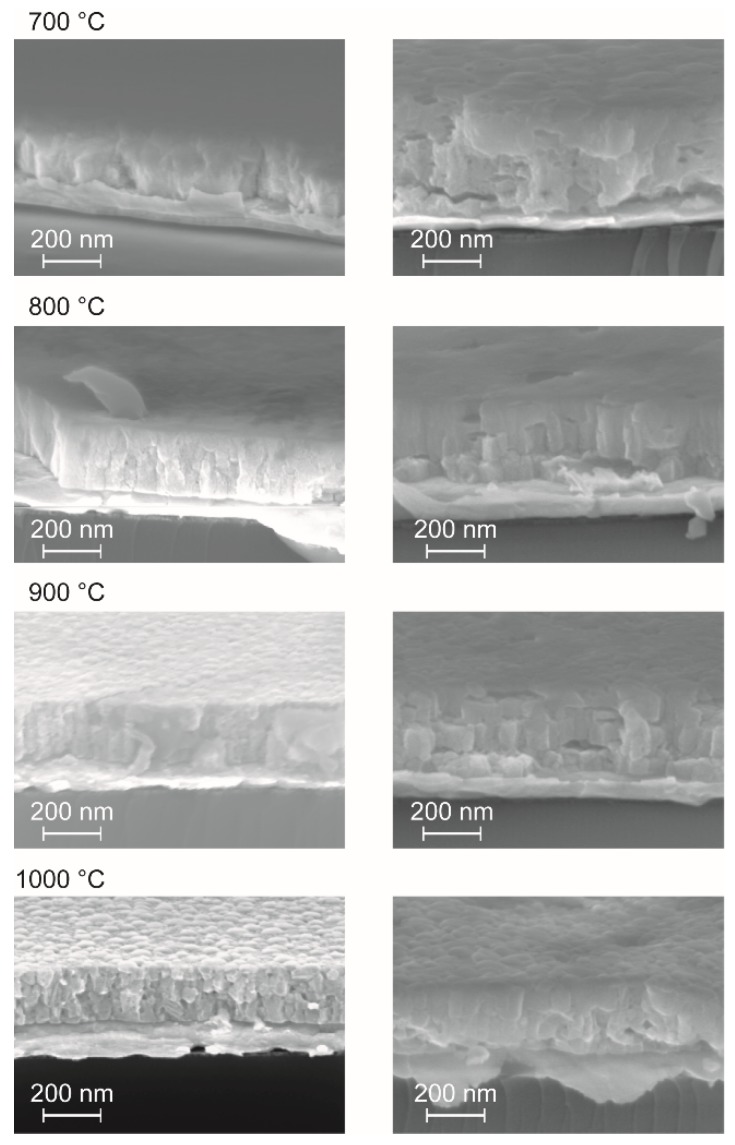
Cross section SEM images from BZY10 films crystallized at different temperatures as indicated. The micrographs shown on the left side stem from five successive coating/annealing cycles of the molecular 0.3 molar *con*-BZY precursor solution, while the SEM images on the right side were recorded from samples prepared from only three successive coating/annealing cycles of the undiluted *µE*-BZY dispersion (c = 0.15 mol·L^−1^).

As an interesting feature, films from both solutions already showed crystallinity after heat treatment at 700 °C, which is ~50 °C lower than expected from the thermal analysis of the bulk precursor gels discussed above. This may be attributed to the substrate surface, which facilitates nucleation in general, and a catalyzing effect of the platinum itself. No secondary phase formation or obvious changes in the XRD pattern are observed if higher crystallization temperatures are applied, but the crystal quality is improved as can be seen from the corresponding cross section SEM images shown in Figure 7. Moreover, a significant decrease of the film thickness with increasing annealing temperatures was observed (Figure 7) which could be attributed to enhanced densification. The most significant decrease in film thickness was observed if the annealing temperature was raised from 700 to 800 °C. Higher temperatures lead to more pronounced crystallites in the films. Interestingly the nanoparticle dispersion derived films show higher thicknesses (e.g., ~270 nm vs. ~200 nm of samples annealed at 1000 °C; Figure 7) although the nominal concentration is only half of those of the conventional precursor solution and less number of coatings are applied (3 vs. 5). This might be explained in terms of different vapor pressures of methanol and propionic acid used as solvents in the two unequal precursor systems, i.e., methanol evaporates faster during spin-coating, leading to a higher material thickness directly after the deposition process.

### 3.2. Inkjet Processing

In preliminary inkjet printing experiments, it turned out that the original stock solution and dispersion yielded non satisfactory results due to blocking of the nozzles and/or bad jetting behavior. Viscosity measurements (*Brookfield DV-II+*) revealed relatively low viscosities, i.e., 2.3 mPa·s for the *con*-BZY10 solution and 0.8 mPa·s for the *µE*-BZY10 dispersion which is a bit out of the range given by the manufacturer of the print head (8–20 mPa·s at jetting temperature). Both data were obtained at 100 rpm corresponding to a shear rate of 132 s^−1^, which are conditions also used in other works dealing with inkjet deposition of CSD precursor solutions [69,71].

Dilution with a suitable solvent is the most obvious and simple way of adjusting the solution properties to the needs of inkjet deposition without massive influencing the phase formation mechanism. The latter has always to be taken into account since in CSD processing, the applied precursor chemistry strongly influences phase and microstructure development [89,90,91]. Hence, care has to be taken if modifiers are used to adjust the viscosity and surface tension are added to the precursor ink. For example, the addition of higher viscosity liquids and/or exchange of organic ligands may increase the organic load, which in turn has to be burned out to form the perovskite thin film. Higher porosity caused by the increased gas evolution during this process may occur. Also in case of alkaline earth metals such as barium, the formation of larger amounts of carbonate compounds should be avoided. Once formed in higher amounts it requires higher temperatures to decompose them quantitatively. Therefore, in this initial work, only dilution with propionic acid in a ratio of 1:1 was applied for the adjustment of the printing properties. The resulting viscosities (1.8 mPa·s, 0.15 M *con*-BZY10; 1.32 mPa·s, 0.075 M *µE*-BZY10) are in the same range as the original stock solution which is nominally still a bit to low but nevertheless yields good printing results (vide infra). In the following *ink-*1 denotes the 0.15 M *con*-BZY10 precursor solution and *ink-*2 is attributed to the 0.075 M *µE*-BZY10 dispersion. Measurements (Data Physics – OCA20) of the surface tension *γ* of the two inks gave very similar values of *γ* = 27.43 ± 0.08 mN/m (*ink-*1) and *γ* = 27.40 ± 0.80 mN/m (*ink-2*).

**Figure 8:**
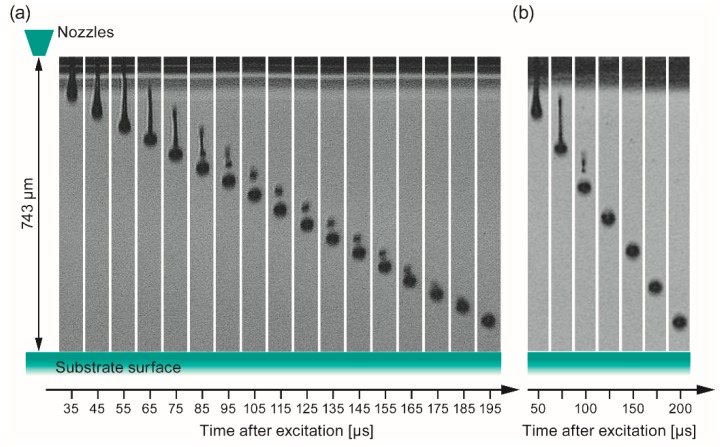
Jetting analysis of the two different inks obtained by dilution of the stock solutions with propionic acid (1:1 vol. ratio) as a function of the elapsed time after triggering the piezoelectric actuator of the nozzle. (**a**) *Ink-*1 (c = 0.15 mol/L, derived from *con*-BZY10); (**b**) *Ink-*2 (c = 0.075 mol/L, derived from *µE*-BZY10). Note, preliminary experiments with methanol as dilution solvent for *µE*-BZY10 did not lead to a reproducible jetting behavior, most likely due to the combination of a low boiling point (i.e., high vapor pressure) and low surface tension of methanol (22.5 mN/m @ 22 °C).

At first the jetting behavior of the two different inks was investigated by the DropView software implemented in the printer system in order to find conditions where isolated spherical droplets without satellite droplets are obtained after being released from the orifice. It is important to choose parameters (printhead excitation, solution formulation, and distance print head – substrate), which could enable formed satellite droplets to merge with the main droplet in order to control the resolution of the printed patterns. Figure 8 shows the result of this analysis. From these stroboscopic images, the droplet’s velocities (*v*) and in-flight diameter (*d*_0_) have been calculated as well. In case of *ink-*1 (Figure 8a) 185–195 µs after the excitation, the intermediately formed satellite droplet merged into one droplet with an estimated diameter (*d*_0_) of 40 µm corresponding to a volume of 33 pl, which is in good agreement with the droplet range given by the manufacturer (25–30 pl). The distance of this single droplet before the impact on the surface was determined to be 90–50 µm that should be sufficiently short to avoid deviation of the droplets from their vertical trajectory by air stream between nozzle and substrate. The speed of the merged droplet was estimated to 3.3 m/s. An investigation of *ink-*2 gave similar results (Figure 8b), i.e., a merged droplet size of *d*_0_ ~ 41 µm corresponding to a slightly higher volume of ~37 pl and an estimated droplet speed of *v* ~ 3.8 m/s.

In order to quantitatively evaluate the present inks concerning their jettability, the typical dimensionless grouping of physical constants, namely Reynolds (*Re*), Weber (*We*), and Ohnesorge (*Oh*) numbers has been applied according to the Equations (1)–(3) [92]:

(1) $$Re=\frac{vd_{\mathrm{Of}}\rho }{\eta },$$

(2) $$We=\frac{v^{2}d_{\mathrm{Of}}\rho }{\gamma },$$

(3) $$Oh^{-1}=\frac{\sqrt{\gamma d_{\mathrm{Of}}\rho }}{\eta }=\frac{Re}{\sqrt{We}}=Z,$$

where *v* is the velocity of the droplets (m/s), *η* is the viscosity (Pa s), *γ* is the surface tension (N/m), *ρ* is the density (kg/m^3^), and *d*_Of_ is a characteristic length (m), which is the diameter of the orifice in the case of DOD print heads.

The parameter Z, which is independent of the droplet velocity and represents the inverse Ohnesorge number (Equation (3)), was introduced by Fromm [93] and later refined by Reis et al. [94] to predict the droplet formation. Hence DOD printing typically takes place in the range of 1 < Z < 10. All obtained data are summarized in Table 1.

Although the obtained Z-values (Table 1) are above the desired upper limit of 10, both inks could be printed in agreement with other reports, which state that printing is possible as long as any satellites formed merge with the main droplet before impact on the surface [53,71]. Overall, for both inks, conditions have been found which are in an acceptable range for the subsequent ink-jet deposition experiments described in the next sections. Since no major chemical modifications by adding modifier molecules have been applied, the obtained properties of the ink-jet derived films can be well compared with those obtained from classical spin coating (cf. Section 3.1).

#### 3.2.1. Individual Printed Dots

**Figure 9:**
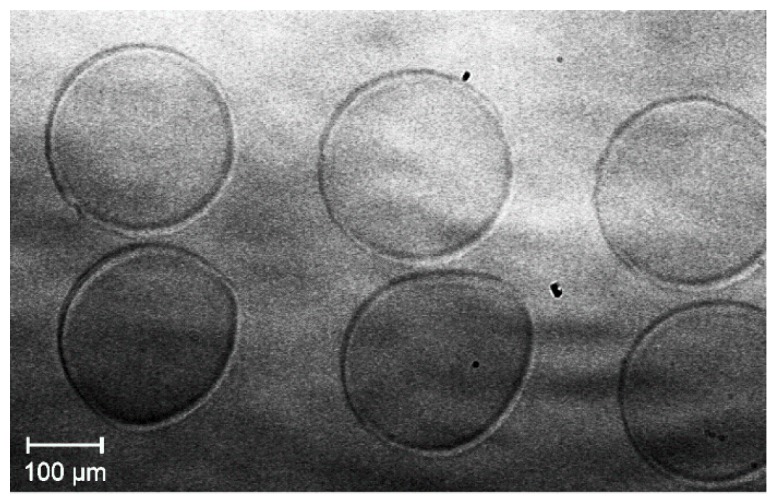
Optical microscopy image of the minimal dot size achievable with the given setup consisting of printer, printhead, and *ink-*1. The nominally printed dots are from left to right 100, 110, and 120 µm in diameter and all show the same printed size (Ø = 220–230 µm) on the substrate.

From the study of printing individual dots on a substrate in the range of 50 to 200 µm nominal diameter, it turned out that the smallest diameter the print system accepts is 100 µm. Thus, PC generated dots in the diameter range of 100–120 µm (Figure 9), for example, are printed as one single droplet, which expands to 220–230 µm upon impacting the substrate surface due to the good wetting behavior of the applied ink described in Section 3.1.2.

For printing dots larger than 150 µm in diameter, two droplets from the same nozzle are deposited onto the surface. Thus, the minimum lateral line dimension printable with this setup is estimated to be 220–230 µm. This has to be taken into account when the desired thin film material pattern is designed at the PC. For the fabrication of full area coated films, an array of dots with suitable distance between the individually deposited drops, which enables merging, has to be printed.

#### 3.2.2. Influence of the Printing Direction

**Figure 10:**
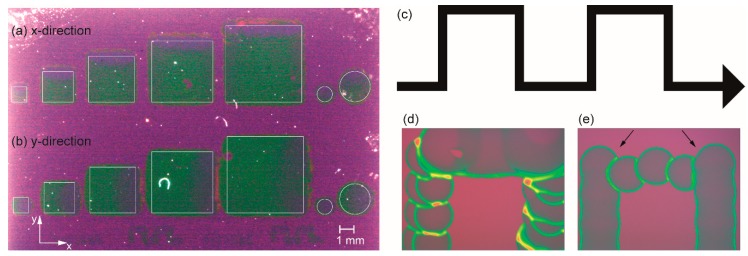
Print results on SiO_2_/Si depending on the main print direction for different features. Left: Photograph showing the results for different areas. The thin white squares (1, 2, 3, 4, and 5 mm^2^) and circles (Ø = 1 and 2 mm) indicate the theoretical areas given by the PC template. (**a**) Printing in the x-direction. (**b**) Printing in the y-direction. Right: Sections of an ∩-type line (schematic part of the template in (**c**); not to scale) nominally printed by 100 µm dots with a resolution of 250 dots per inch (DPI). (**d**) Printing in the x-direction (**e**) Printing in the y-direction. However, there is a pronounced positional inaccuracy at the edges (arrows) which can be attributed to the movement of the as-deposited drops in the y-direction. The slightly shifted dots in the upright lines of photomicrograph (**d**) may be explained by the acceleration of the as-deposited, still flowable dots caused by the incremental movement in the y-direction before the next droplet is printed.

The printing direction as a further factor has to be considered as well. The present printer setup offers a number of different print directions that are realized by different moveable parts, such as the x-direction, by movement of the print head along the x-axis, as well as the y-direction by movement of the substrate perpendicular to this. The difference is related to the fact which part of the printer setup is in motion while jetting, and linked to that a different number of nozzles is used. This means that during printing in the y-direction, the substrate is moved while the droplets, generated by a number of simultaneously jetting nozzles, arrive on the substrate surface. On the other hand, printing in the x-direction involves the usage of only one jetting nozzle while moving along the x-axis. After the line is finished, the substrate is moved incrementally in the y-direction, followed by printing the next line. To figure out the impact of this parameter, the same patterns have been printed by printing only in the x-direction, and in another experiment only in the y-direction (Figure 10) by using *ink-*1.

Provided the requests for structural accuracy tolerate deviations in the µm range both methods yield viable results. The slightly higher accuracy, i.e., better defined areas, are obtained from the x-direction (Figure 10a). However slightly better homogeneity of the coating is obtained from the y-direction method (Figure 10b). This can be explained in terms of a better merging of the individual droplets due to more simultaneously deposited drops (more nozzles jetting). There is less time for evaporation of solvent, leading to better flowability compared to the dots deposited from single nozzle operation in the x-direction mode. The lines observed in the printed areas of the latter (Figure 10a) may be explained by the acceleration during the incremental movement of the substrate in y-direction after printing a line in x-direction. This effect is easier to see in case of printing thin ∩-type single lines (Figure 10c) as shown in the micrographs of one “∩” in Figure 10d,e. Although there is also some deviation, the overall quality of the line shape printed in the x-direction (Figure 10d) is slightly higher compared to that obtained from the y-direction, as shown in Figure 10e. In summary, both methods enable the patterned deposition with the given setup and both inks are essentially suitable for ink-jet deposition. Nevertheless, further modification of the inks in order to optimize the resolution of the printed structures is required and will be part of further work. Based on the present achievements, the formation and properties of dense, full-area, and ink-jet deposited BZY10 films are studied first in the following sections, since this is an important prerequisite for using such proton conducting membranes in future intermediate temperature operating electrochemical devices.

### 3.3. Full Area Ink-Jet Printed BZY10 Films

**Figure 11:**
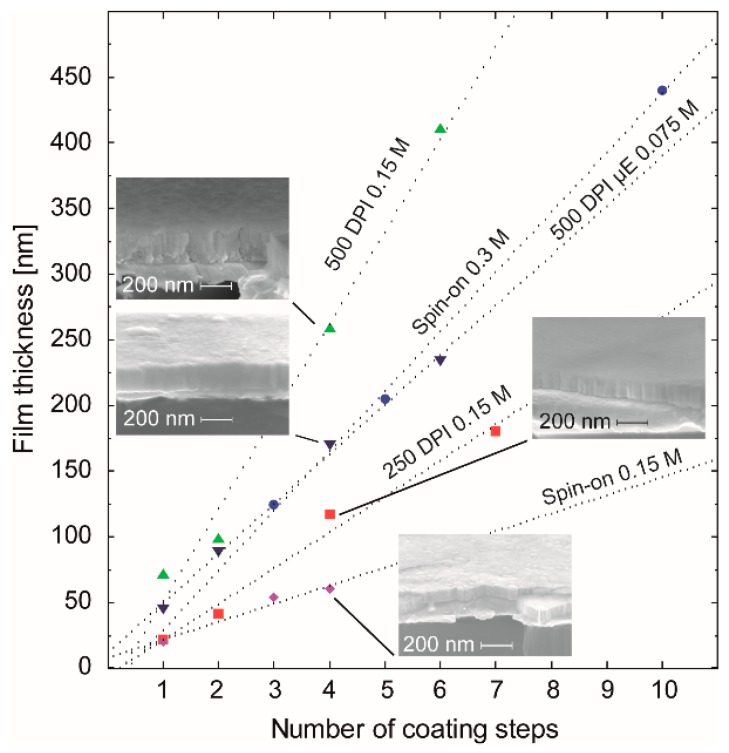
Correlation of the film thickness with the number of coating steps using different coating conditions for *ink-*1 and *ink-*2 (*µ*E), namely spin coating at 3000 rpm (Spin-on 0.15 M) and ink jet printing with two different resolutions (250 DPI 0.15 M, 500 DPI 0.15 M, and 500 DPI µE 0.075 M). The corresponding behavior of films obtained from the spin coated original con-BZY stock solution was plotted as well. The cross section SEM images of the four insets show the microstructure of films produced by four coating steps using *ink-*1 and *ink-*2.

Both precursor inks have been used for ink-jet deposition experiments on 1 × 1 inch^2^ platinized silicon wafers aiming to develop full area dense electrolyte films. Focused on the use of *ink-*1 two different print resolutions, namely 250 DPI and 500 DPI, have been applied corresponding to a printed spacing between two drops of nominally 101.6 µm and 50.8 µm, respectively. The diameter of the nominally printed dots was set to 100 µm. Due to the ~2.3 fold larger effectively formed dot area (cf. Section 3.2.1) already present in the case of a lower resolution, a good merging of the as-deposited droplets can be expected. Application of the larger resolution results in higher quantity of deposited material per area and thus to larger film thickness per printing step (Figure 11). For comparison, the thickness evolution of *ink-*2 in case of the 500 DPI print resolution was investigated as well and plotted in Figure 11.

From the analysis of the dotted regression lines in Figure 11, the thickness gain per coating step (nm/#) has been determined to be 27 nm/# (250 DPI), 70 nm/# (500 DPI), and 14 nm/# (Spin-on) for *ink-*1 (0.15M). For the spin coated 0.3 M stock solution, 46 nm/# was obtained. A calculation of this parameter for *ink-*2 (0.075M) deposited with a resolution of 500 DPI gave a value of 38 nm/#. Thus, compared to spin-on deposition, the inkjet process enables the same film thickness from the same concentration with a significantly lower number of coating/annealing cycles, which may be an additional benefit of this technique.

In order to investigate the electrochemical properties of ink-jet printed BZY10 films, full area coated films from the two different inks (molecular and nanoparticle based) have been prepared on sapphire. Six printing/1000 °C-annealing steps using *ink-*1 and *ink-*2 yielded fully crystalline BZY10 films denoted as BZY*_ink-_*_1_ and BZY*_ink-_*_2_ with a thickness *d* of 420 and 228 nm, respectively. The thickness was estimated from the thickness increase per coating as determined from Figure 11. XRD measurements proved the films crystallinity and the analysis of the diffractograms yielded a lattice constant of 4.180 Å for the BZY*_ink-_*_1_ film and 4.193 Å for the BZY*_ink-_*_2_ film. Hence, both BZY10 films grown on sapphire show a higher lattice constant compared to those fabricated on platinized silicon, which is closer to the bulk value but still a bit too low. Obviously, there is less compressive stress in the BZY films deposited on the sapphire substrate. The considerable difference of the lattice constants between both films on sapphire point to different nucleation and growth behavior during crystallization treatment. This in turn may be related to the different precursor systems. While *ink-*1 is a molecular precursor, which at first has to decompose and form a layer of an amorphous metal oxide mixture, *ink-*2 consists already of preformed amorphous BZY10 nanoparticles that form a deposited layer. Both layers have to transform into the crystalline layer through a nucleation and growth process. However in case of the *ink-*1 derived amorphous layer, the substrate/film interface with the accompanied clamping of the film may play a larger role as in the case of the preformed nanoparticles derived from *ink-*2, which leads to a more independent crystallization process in the latter case and thus to crystallites with a lattice constant closer to the bulk value.

**Figure 12:**
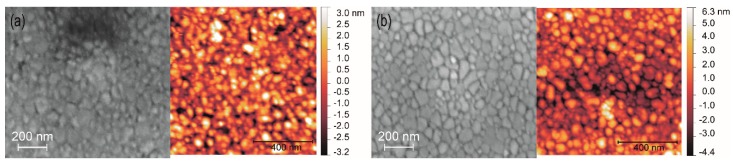
Top-view SEM images (left) and corresponding AFM images (right) of the ink-jet prepared samples on sapphire substrates (**a**) BZY*_ink-_*_1_ film and (**b**) BZY*_ink-_*_2_ film.

From the XRD data, the crystallite sizes of the two different BZY10 films were estimated using the Scherrer formula with the shape factor of spheres (K = 0.89). The required FWHM (full width at half maximum) values were obtained from the PANalytical program “X’Pert High Score” and crystallite sizes of 42 nm for the BZY*_ink-_*_1_ and 44 nm for the BZY*_ink-_*_2_ film were calculated. They are in good agreement with the estimated value of ~50 nm obtained from the top view SEM and AFM images (Figure 12). Both surfaces show similar grains and from the comparison of the two SEM images in Figure 12 one may deduce a slightly lower density and higher roughness of the BZY*_ink-_*_2_ film, which is confirmed by the RMS values obtained from the corresponding AFM measurements (Figure 12a right image: 1.3 nm RMS, Figure 12b right image: 1.8 nm).

### 3.4. Electrical and Electrochemical Characterization

At first, permittivity measurements on exemplary BZY10 samples in the thickness range of around 100 nm were performed. For these measurements metal-insulator-metal structures were used which were prepared from the films fabricated on platinized silicon wafers by depositing square 0.025 mm^2^ Pt-top-electrodes (*d* = 100 nm) by sputtering and lift-off processing. The obtained room temperature permittivities (50 mV, 1 kHz), namely 33 (*con-BZY*, spin-on, *d* = 124 nm), 39 (*ink-*1, 500 DPI, *d* = 98 nm), and 37 (*ink-*2, 500 DPI, *d* = 95 nm) compare well with values in the literature for CSD derived BZY thin films (41 [95]) and differently processed bulk BZY samples (~52–98). Although in the latter bulk case the data were obtained from measurements at higher temperatures (100 °C–350 °C) [31,96,97], they were still in the same order of magnitude. Overall, the obtained permittivities suggest that the dielectric properties of the present BZY10 thin films were independent of the deposition process and that fundamental material integrity is given.

**Figure 13:**
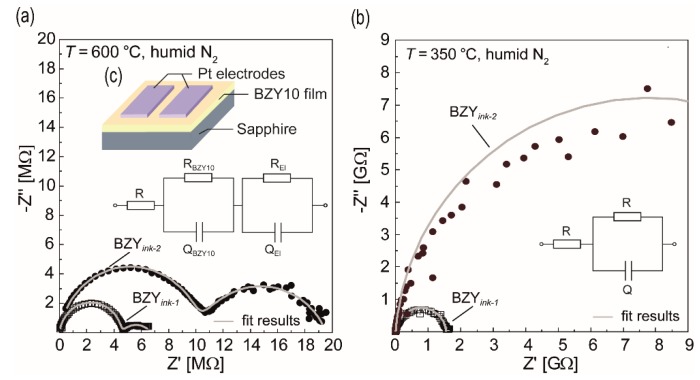
Impedance spectra of the two ink-jet printed BZY10 films BZY*_ink-_*_1_ (molecular based) and BZY*_ink-_*_2_ (nanoparticle based) measured at different temperatures by in-plane mode. Shown are also the results of the fitted semicircles (grey lines), which have been obtained according to the schematically depicted equivalent circuits. (**a**) 600 °C in humidified nitrogen. (**b**) 350 °C in humidified nitrogen. The inset (**c**) shows the sample design used for the in-plane measurement of the impedance.

In order to prove the fundamental functional response with regard to proton conduction, high temperature electrochemical impedance spectroscopy (HT-EIS) was applied to the two different ink-jet printed BZY10 samples described above (BZY*_ink-_*_1_ and BZY*_ink-_*_2_). The in-plane measurements (cf. Figure 13c) have been performed in a temperature range between 600 °C and 150 °C in descending steps of 50 °C. Figure 13 shows the Nyquist plots of the two samples at two exemplarily selected temperatures.

At 600 °C, both films show two semicircles in the impedance spectra, which could be fitted by an equivalent circuit consisting of a series connection of *R* and two *RQ* elements (Figure 13a). *Q* represent constant phase elements and *R* the resistance. The resistance *R* was added to the model to consider a possible contribution of the Pt contact wiring. For the high frequency semicircles, *Q* values in the pF range (~1.7 pF/*ink-*1, ~4 pF/*ink-*2) have been observed. As reported in the literature for the case of in-plane EIS measurements of thin electrolyte films grown on substrates such as sapphire [98,99], it can typically not be differentiated between grain and grain boundary contributions due to the influence of the stray capacitances. Thus, this semicircle is attributed to the total electrolyte film response (*R*_BZY10_*Q*_BZY10_), i.e., combined bulk and grain boundaries. The second, low frequency semicircle is typically attributed to the electrode polarization and may also stem in the present work from the electrodes (*R*_El_*Q*_El_) although the *Q* values (~38 nF/*ink-*1, ~3 nF/*ink-*2) are not in the typical range (~10^−6^ F). When decreasing the temperature, this low frequency contribution increasingly vanishes below 400 °C, meaning only one high frequency semicircle remains visible (cf. Figure 13b). Hence, it is concluded that the only information which can be gathered from EIS in the present study is the resistance of the corresponding BZY10 film. From the resistance data (*R*_BZY10_) and the geometry of the samples, the total conductivities *σ* were calculated according to:

(4) $$\sigma =\frac{1}{R_{\mathrm{BZY}10}}\cdot \frac{D}{A},$$

with *D* relating to the distance between the two Pt electrodes and *A* corresponding to the area obtained from the length of the electrodes times the corresponding film thickness *d*. For the two exemplary temperatures conductivities of *σ* = 4.0 × 10^−6^ S/cm (350 °C) and *σ* = 1.3 × 10^−3^ S/cm (600 °C) for BZY*_ink-_*_1_, and *σ* = 4.5 × 10^−7^ S/cm (350 °C) and *σ* = 6.9 × 10^−4^ S/cm (600 °C) for BZY*_ink-_*_2_ have been obtained by applying Equation (4). These values are in a comparable range to other polycrystalline films described in the literature, e.g., ~10^−6^ S/cm at 350 °C and ~ 1.6 × 10^−4^ S/cm at 600 °C (*d* = 6 µm) reference [22], or ~4.6 × 10^−4^ S/cm at 600°C (*d* = 480 nm) thick reference [35] which, however, have been prepared by PLD.

**Figure 14:**
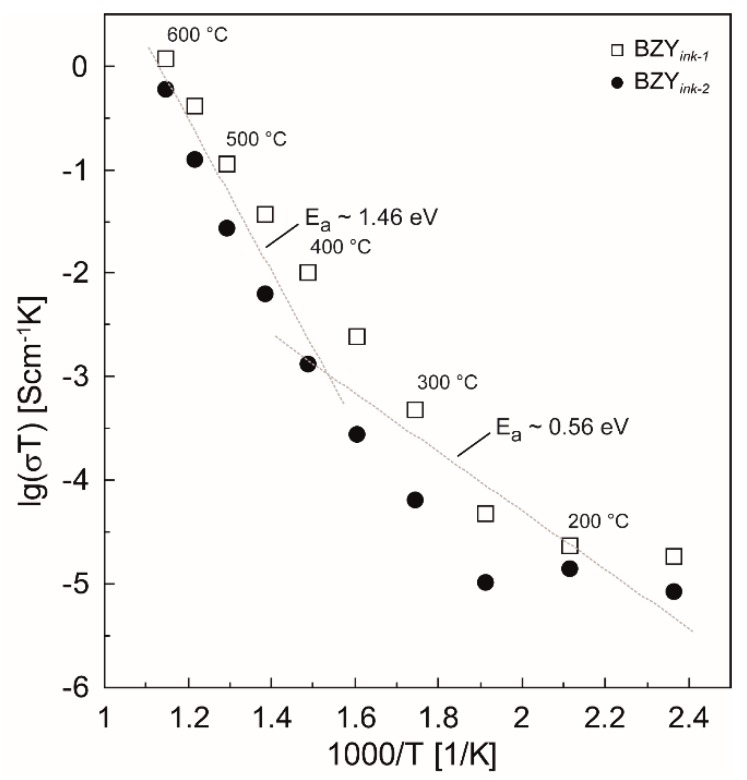
Arrhenius Plot of the two different samples BZY*_ink-_*_1_ and BZY*_ink-_*_2_ in the temperature range from 150 to 600 °C. The dotted grey lines are a guidance for the eyes indicating two different regimes of activation energies. The slopes of these grey lines represent the averaged E_a_ values obtained from the fitted graphs in the two regions i.e., below approximately 400 °C and above.

The results of the temperature dependent impedance spectroscopy for the two different films are shown in the Arrhenius plots of Figure 14. There is a clear change of the slopes of the logarithmized conductivities in the Arrhenius plots around 350 °C, which indicates a change of the preferred charge carrier transport in the material. Below 400–350 °C, an activation energy of ~0.56 eV was found, which indicates proton conduction in agreement with the literature. At higher temperatures, the dehydration of the material takes place, leading to possible contributions of oxygen vacancies and holes that possess higher activation energies (Figure 14, T > 400 °C). Based on the present experiments, however, this issue can finally not be clarified. Nevertheless, it is thermodynamically self-evident that the dehydration takes progressively place in this temperature range [22,82,83].

Overall, the present ink-jet printed BZY thin films show conductivities comparable to thin films on similar substrates prepared by gas phase methods, but lower than bulk BZY ceramics. In agreement with literature known polycrystalline films prepared by PLD, this is attributed to the smaller grains and hence to a larger number or proton transport blocking grain boundaries.

## 4. Conclusions

Ink-jet printing of a modified BZY10 spin coating solution and a microemulsion based BZY10 nanoparticle dispersion has been successfully demonstrated by means of crystalline and dense BZY10 films. Therefore at first, the decomposition and phase formation behavior of the two different precursor systems have been studied in order to find suitable processing conditions. It turned out that both precursor systems already yield crystalline BZY10 films at 700 °C but increasing temperatures led to higher crystallinity. Thus annealing at 1000 °C was found to be optimal with regard to the crystallinity of BZY and stability of the utilized substrates. The investigation of the rheological properties of the two precursor solutions and an appropriate, moderate modification by dilution with propionic acid enabled printable inks. Both types of the resulting crystalline films showed averaged permittivities of ~36, which compares well to the data given in the literature. The HT-EIS analysis of samples printed on sapphire revealed an activation energy of ~0.56 eV in the temperature range below 400–350 °C, which pointed to proton conduction, as expected for this material. The corresponding relatively low conductivities were around 10^−6^ S/cm (350 °C) and compared well with other polycrystalline thin films prepared by the more expensive PLD technique. Increasing the measuring temperature led to increasing conductivities up to ~10^−3^ S/cm (600 °C). Comparatively small grain sizes of ~50 nm were found in the films and may be one reason for the observed conductivities, which were lower than those of the respective bulk material.

Patterning was essentially possible and may be further optimized by changing the rheology of the inks in order to adjust the contact angle and spreading behavior. The method is ideally suited for the fabrication of integrated proton conducting layers on membranes made from micromachining, thereby enabling the realization of miniaturized proton conducting fuel cells or sensor devices working at intermediate temperatures. If combined with suitable inks for the electrode material, such as (La_1−x_Sr_x_)(Co_1−y_Fe_y_)O_3−δ_ for the cathode, complete patterned thin film stacks can be additively manufactured with a reduced loss of precursor material and number of patterning steps. Further work is in progress to evaluate these possibilities.

## Figures and Tables

**Table 1:** Fluid properties of the two different BZY10 inks used in the present study.

Ink Type	*v* (m/s) ^1^	*d*_Of_ (m)	*ρ* (kg/m^3^)	*η* (Pa s) ^2^	*γ* (N/m)	*Re*	*We*	*Oh^−1^ = Z*
1 (con-BZY)	3.3	35 × 10^−6^	1001	1.80 × 10^−3^	27.43 × 10^−3^	64.23	13.91	17.22
2 (µ-E-BZY)	3.8	35 × 10^−6^	946	1.32 × 10^−3^	27.40 × 10^−3^	95.32	17.45	22.82

^1^ The droplet’s velocity (*v*) was calculated from the stroboscopic images. ^2^ The viscosities were measured at a shear rate of 132 s^−1^.
